# Infection surveillance measures during the COVID-19 pandemic in Germany

**DOI:** 10.3205/dgkh000398

**Published:** 2021-09-14

**Authors:** Antonia Milena Köster, Anna Bludau, Sanja Katharina Devcic, Simone Scheithauer, Amelia Aquareta Mardiko, Reiner Schaumann

**Affiliations:** 1 Institute for Infection Control and Infectious Diseases, University Medical Center Göttingen, Göttingen, Germany

**Keywords:** scoping review, outbreak, infection protection, public health, testing, contact tracing, application

## Abstract

**Introduction:** To address the question as to which infection surveillance measures are used during the ongoing COVID-19 pandemic in Germany and how they differ from pre-existing approaches.

**Methods:** In accordance with the systematic approach of a scoping review, a literature search was conducted in national and international medical literature databases using a search string. The search in the databases was limited to the period from 01.01.2000 to 15.11.2020 and has been subsequently completed by hand search until 08.03.2021. A hand search, even beyond 15.11.2020, seemed necessary and reasonable, since due to the dynamics of the ongoing COVID-19 pandemic, a large number of articles and regulations are being published very quickly at short notice.

**Results:** The literature search resulted in the following number of hits in the databases listed below:

- PubMed: 165 articles
- Cochrane: 1 review and 35 studies
- Web of Science: 217 articles
- Robert Koch Institute: 49 articles

Thus, a total of 467 hits were identified, with a total of 124 hits being duplicates. From these, 138 articles were considered relevant to the COVID-19 infection surveillance situation in Germany based on established criteria. After reading the full texts, 92 articles and websites were ultimately included in the scoping review.

**Discussion:** Many of the lessons learned from previous outbreaks seem to have been implemented in the infection surveillance measures during the ongoing COVID-19 pandemic in Germany. Most of the changes compared with previous measures were based on technological streamlining of existing procedures and changes and more inclusion of the population in different infection surveillance measures.

## Zusammenfassung

**Einleitung:** Es wird der Frage nachgegangen, welche Maßnahmen zur Infektionsüberwachung während der aktuellen COVID-19-Pandemie in Deutschland eingesetzt werden und wie sie sich von bereits bestehenden Ansätzen unterscheiden.

**Methoden:** Entsprechend der systematischen Vorgehensweise eines Scoping Reviews wird eine Literaturrecherche in nationalen und internationalen medizinischen Literaturdatenbanken mittels Suchstring durchgeführt. Die Suche in den Datenbanken ist auf den Zeitraum vom 01.01.2000 bis zum 15.11.2020 begrenzt und anschließend per Handsuche bis zum 08.03.2021 ergänzt. Eine Handsuche, auch über den 15.11.2020 hinaus, erscheint notwendig und sinnvoll, da aufgrund der Dynamik der laufenden COVID-19-Pandemie sehr kurzfristig eine große Anzahl von Artikeln und Verordnungen veröffentlicht werden.

**Ergebnisse:** Die Literaturrecherche hat die folgende Anzahl von Treffern in den unten aufgeführten Datenbanken ergeben:

- PubMed: 165 Artikel
- Cochrane: 1 Review und 35 Studien
- Web of Science: 217 Artikel
- Robert Koch-Institut: 49 Artikel

Somit sind insgesamt 467 Treffer identifiziert worden, wobei insgesamt 124 Treffer Duplikate sind. Anhand festgelegter Kriterien werden 138 Artikel als relevant für die COVID-19-Infektionssurveillance in Deutschland eingestuft, und nach dem Lesen der Volltexte schließlich 92 Artikel und Webseiten in den Scoping Review aufgenommen.

**Diskussion:** Viele der Maßnahmen, die aufgrund früherer Ausbruchsgeschehen bekannt sind, scheinen auch bei der jetzigen Infektionsüberwachung während der laufenden COVID-19-Pandemie in Deutschland umgesetzt worden zu sein. Die meisten Änderungen im Vergleich zu früheren Maßnahmen betreffen technologische Neuerungen sowie eine stärkere Einbeziehung der Bevölkerung in die verschiedenen Maßnahmen zur Infektionsüberwachung.

## Introduction

Pandemic surveillance strategies include population-based, pathogen-based, and patient-based surveillance, and appropriate methods of active or passive data collection are employed to determine efficiency and cost-effectiveness [1]. In order to gain information and guidance, national and international publications are generally consulted for pandemic data and interventions, including websites and materials on global COVID-19 surveillance and comparative World Health Organization (WHO) and EU results. In addition, lessons learned from three previous significant nation-wide disease outbreaks in the 21^st^ century, such as Severe Acute Respiratory Syndrome (SARS), Swine Flu (H1N1), and Enterohemorrhagic *Escherichia coli* (EHEC) are considered in the analysis of the COVID-19 pandemic. In this respect, countless proposals, statements and publications on the current COVID-19 pandemic are now available [2]. In addition to the continuously updated publications of the WHO, there are comprehensive summaries of internationally available information on this topic [3]. It is conspicuous that classic surveillance aspects are increasingly being expanded and discussed under the concept of “preparedness”, as is clear, for example, in the overview “COVID-19 strategic preparedness and response” [4]. Similarly, methodological and specific questions on surveillance are summarized and discussed internationally [5]. Further examples are environmental surveillance, public health surveillance and the protection of health professionals [6], [7], [8]. A consensus statement issued in October 2020 by the WHO Strategic and Technical Advisory Group for Infectious Hazards, in which Germany is represented by the Robert Koch Institute (RKI), points the way forward. Entitled “Living with the COVID-19 pandemic: act now with the tools we have“ [9]. The statement focuses on overarching strategic recommendations. This includes a compilation of the essential tools and strategies of surveillance, prevention, and response [9].

In the European context, further and specific summary analyses and assessments are available. Of particular note is the collaborative initiative of the WHO *Regional Office for Europe*, *European Commission*, and *European Observatory on Health Systems and Policies*, which developed the COVID-19 Health Systems Response Monitor (HSRM), a framework for national reports, thus providing opportunities for European assessments and comparative analyses. Key findings are summarized in a thematic issue of Eurohealth [10]. Most relevant to surveillance issues are the contributions of Rajan et al. and Hernandez-Quecedo et al. [11], [12].

These international and European websites and materials provide an overview of the available information on COVID-19 surveillance. In many cases, the materials have been compiled using consensus-based methodological procedures and are based on information and research from internationally recognized experts. The international perspective combined with the highly aggregated and comparative materials provide orientation in a still very dynamic and often even confusing research situation. However, since they focus on overarching issues and thus make highly generalized statements, little attention has so far been paid to specific questions and concrete aspects of implementation of surveillance, taking into account the actual situation in the respective countries and their health care systems.

For the reasons mentioned here, an ad hoc literature search was conducted in national and international medical databases using the systematic approach of a scoping review to present specific aspects on the situation of COVID-19 infection surveillance in Germany during the current pandemic.

## Ad hoc literature search

With respect to infection surveillance in a pandemic situation, an ad hoc literature search should be conducted in the context of globally used strategies, objectives, units of measurement of surveillance, aggregation methods and presentation of measured endpoints and influencing factors, as well as user-friendly and interactive web tools to illustrate and make available the surveillance information thus obtained [1]. Thus, in order to do justice to this project, a literature search was carried out at the national and international level, including a review of websites and materials on global COVID-19 surveillance and EU-comparative results. In accordance with the systematic approach of a scoping review, a literature search was conducted in national and international medical literature databases using the search string listed in Table 1 (Tab. 1). The database search was limited to the period from 01.01.2000 to 15.11.2020. The final query was performed on 15.11.2020 and was subsequently completed by hand search up to 08.03.2021. A hand search, even beyond 15.11.2020, seemed necessary and reasonable: due to the dynamics of the ongoing COVID-19 pandemic, a large number of articles and regulations are published very quickly at short notice. English search terms are used in the international databases, and these were supplemented with German search terms in the national databases when necessary. Relevant segments were COVID-19 and previous pandemics and/or emerging respiratory infections, such as MERS-CoV, SARS, H1N1, swine flu, and surveillance and Germany. Within these segments, synonyms and topic-like terms acted as search terms.

The literature search yielded the following number of hits in these databases:

- PubMed: 165 articles
- Cochrane: 1 review and 35 studies
- Web of Science: 217 articles
- Robert Koch Institute: 49 articles

The query in the German databases “*Elektronische Zentralbibliothek*” and “*Deutsche Nationalbibliothek*” did not yield any additional results. That is, a total of 467 hits were identified, with a total of 124 hits being duplicates. After reviewing the abstracts, 138 articles were considered relevant to the COVID-19 infection surveillance situation in Germany, based on established criteria and, after reading the full texts, 92 articles and websites were ultimately included in this review. 

The criteria are:

1. No vaccination surveillance
2. No exclusive tool surveillance
3. No exclusive test or test method surveillance
4. No exclusive management and strategy surveillance
5. No exclusive therapy surveillance, whether pharmacological or nonpharmacological
6. Inclusion of articles related to Europe, defined as EU, EEA, EFA including the UK as a past member of the EU, and at least also to Germany
7. Exclusion of articles titled with the word “estimations”, “predictions”, and “simulations”

The articles were used to obtain information and references on the applied infectious disease surveillance strategy in Germany and to present these as comprehensively as possible in this review. Based on this, a German-language definition of surveillance was extracted for further discussion. According to the definition of the CDC Atlanta [13] and the London School of Hygiene [14] surveillance is 

“the ongoing systematic collection, analysis, and interpretation of health data, essential to the planning, implementation, and evaluation of public health practice, closely integrated with the timely dissemination of these data to those who need to know.”

The German-language extracted definition of the above-mentioned definition of surveillance is listed below (translation by the authors):

‘Surveillance im Gesundheitswesen ist die fortlaufende, systematische Sammlung, Analyse und Interpretation von Gesundheitsdaten der Bevölkerung. Sie ist unerlässlich für die Planung, Umsetzung und Bewertung von öffentlichen Maßnahmen für die Gesunderhaltung der Bevölkerung. Surveillance im Gesundheitswesen ist auch eng verbunden mit der rechtzeitigen Weitergabe dieser Daten an diejenigen, die hiervon Kenntnis haben müssen.’

This working definition is generally applied to infectious diseases during infection surveillance. The prerequisite for this is systematic recording as well as methodical communication with stakeholders in order to ensure an assessment of health threats and the initiation of possible control measures. Surveillance strategies and surveillance tools are among the central pillars of COVID-19 pandemic control, which will be taken into account in the following discussion of the results of the literature search. This article presents the structural framework provided by the Infection Protection Act, provides insights gathered from previous outbreaks in the 21^st^ century, describes the current outbreaks, and provides a description of selected infection surveillance measures, i.e., testing, contact tracing and technological applications. 

## Infektionsschutzgesetz (Infection Protection Act)

The “Infektionsschutzgesetz1471” (IfSG) became effective for the first time on July 20, 2000, in Germany, and has been subject to repeated updates, additions and amendments ever since. The aim is the prevention of communicable diseases (§ 2 para. 3 IfSG) as well as the early detection of infections and the prevention of their further spread (§1 IfSG). The primary tasks of the Robert Koch Institute (RKI) include scientific research, epidemiological and medical analysis and evaluation of diseases with a high degree of risk, high prevalence or high public or health policy importance. The RKI is based in the Federal Institute of Health within the Federal Ministry of Health (Bundesministerium für Gesundheit [BMG]). In this context, “the Robert Koch Institute (RKI) is the government’s central scientific institution in the field of biomedicine” [15]. The core tasks of the RKI are the detection, prevention and control of diseases, especially infectious diseases for the development of health policy decisions. In an epidemiological situation of national importance (§ 5 para. 1 IfSG), the RKI is responsible for cooperation and information exchange between countries (§ 5 para. 7 IfSG). When reportable COVID-19 cases occur, senior physicians or physicians who have identified the disease are primarily required to report the case (§ 8 Abs. 1 IfSG). In the case of a named person, the contact details of the infected person must be submitted to the responsible health authority within 24 hours (§ 9 para. 3 IfSG). In the case that a person is not named, both the contact details of the reporting person and the contact details of the person concerned must be reported to the relevant health authority within 24 hours (Section 10 (1) IfSG). The relevant health authority completes the reported data and evidence, and compiles them before they are forwarded to the relevant state authority. The data are transmitted on the following weekday to the RKI (§ 11 para. 1 IfSG), where various case definitions are available for the evaluation of suspected cases, cases of illness or death (§ 11 para. 2 IfSG).

In addition, if an adverse event occurs in connection to a vaccination the public health department responsible reports the most important information to the competent state authority in accordance with Section 2 (11) IfSG, which forwards it to the Paul Ehrlich Institute (PEI) and/or the Federal Institute for Vaccines and Biomedical Products (Section 11 (4) IfSG). These measures are intended to ensure the prevention of transmissible infections and to prevent and contain their spread. In addition, a high level of reliability and accuracy in contact tracing must be ensured [16], [17], [18], [19]. 

## Previous nation-wide disease outbreaks

In the 21^st^ century, three significant nation-wide disease outbreaks occurred in Germany prior to the current COVID-19 pandemic: Severe Acute Respiratory Syndrome (SARS), Swine Flu (H1N1), and Enterohemorrhagic Escherichia coli (EHEC) [20], [21], [22], [23], [24], [25]. Experience with these outbreak events, and the expectation of further such events [26], [27], should be considered when analyzing the current management and surveillance of the COVID-19 pandemic [28], [29], [30].

The SARS surveillance system was established by the RKI in March 2003 directly after the announcement of the first cases of infection in Germany. The results of a scientific analysis of the system showed that data transmission and capacity of the system needed to be improved for the future [22]. An up-to-date, electronic information system is necessary for the immediate transmission of reports from the health authorities to the RKI. In addition, an expansion of the capacity of the surveillance system to process large amounts of data is necessary. Finally, there is a need for advance clarification of the case definition as well as criteria for mandatory reporting [22]. As a result of surveillance evaluation, after the initial emergence of pandemic H1N1 influenza in 2009 to 2010, it was found that area-wide hospital and mortality surveillance tools were lacking at the onset of the pandemic [24], [31], [32]. In addition, recording systems that validly document data on mortalities, clinical courses, or care utilization were lacking. Many public health departments were temporarily overburdened by individual cases of infection surveillance in accordance with the IfSG. This indicated the importance of having a more flexible IT system for surveillance that can be adapted to the specific information needs and the respective pandemic situation. Furthermore, hospital surveillance systems should be implemented and established to document important information about the burden on hospitals as well as severe disease progression [28]. To improve the communication strategy, a future establishment of modern, digital communication systems was called for at that time [24], [31], [32].

The world’s largest outbreak of EHEC to date occurred nation-wide from May to July 2011, with the EHEC serotype 0104:H3 in Germany [33]. Although the disease is not a respiratory infectious disease, certain similarities to the current pandemic situation exist in terms of surveillance management. According to Krause et al., the need for sentinel surveillance exists when reporting requirements for specific information about a disease are limited [21], [24]. In this respect, the motivation to participate in such survey systems should be regularly promoted among practicing physicians, hospitals and laboratories. Finally, mortality surveillance should be established immediately to enable regular reporting of deaths by the RKI and state authorities [23]. 

## Outbreak events caused by the infectious disease COVID-19

Collection and analysis of data on COVID-19 cases as well as outbreaks are important steps to better understand infection events and circumstances. “Gesundheitsämter” (GA; public health offices) in Germany are an important component for contact tracing and follow-up, as well as identification of sources of infection. As recently as the end of April 2020, the number of GA teams across Germany was increased, and a financial package to digitize GAs was approved [34]. 

Section 11 of the IfSG specifies the information that GAs are allowed to transmit to the appropriate state authorities and the RKI after the survey. This includes the probable route of infection, the probable risk of infection, recognizable affiliation with a cluster of illnesses, and the location at which the infection probably occurred [35]. Via the software SurvNet, probable chains of infection or cases that may be epidemiologically related to each other are combined to form an “outbreak” and transferred in bundled form to the state authorities and the RKI [35]. This is a figure which includes only laboratory-confirmed COVID-19 cases according to the RKI reference definition and outbreaks with at least two cases [35]. The RKI publishes information on the current outbreak situation once a week in a situation report [36]. Smaller outbreak events with two to four cases, which are particularly common in private households, account for a large proportion of outbreaks. Larger outbreaks (≥10 cases) occurred primarily in nursing homes and homes for the elderly, hospitals, refugee/asylum seekers’ shelters, at schools, and in occupational settings [35], [37], [38], [39]. One of the largest outbreaks occurred in June 2020 at a meat-processing plant in Gütersloh County, with a total of 2,117 COVID-19 cases among plant employees and persons in their environment [40]. By increasing the seven-day incidence, these outbreaks may lead to initiations of restrictive measures. However, identifying outbreak events is not without limitations, as outbreaks in anonymous groups of people, such as on public transportation, are more difficult for GAs to detect and track, and attribution to a single infection area is not always possible. Furthermore, GAs lack the capacity to follow up on outbreak information in detail during the peak phases of the pandemic.

The SORMAS software developed for this purpose could expand capacity [41]. All 400 GAs should have been equipped with SORMAS by the end of February 2021 [42]. However, only about 80-150 offices use the software, that is justified by a lack of functions and interfaces as well as a costly conversion, or because changes in workflows during the pandemic are seen as a hindrance [42]. With respect to GAs, the continued lack of widespread implementation of the software shows that they are still overwhelmed. 

## Testing as a measure of infection surveillance

Germany already had a powerful laboratory infrastructure at the beginning of the pandemic and thus a relatively large testing capacity for the detection of SARS-CoV-2 [43]. In the first weeks of the pandemic, more than 1.7 million tests were performed. The positivity rate of these tests was approximately 10%. On the one hand, this shows high availability for suspected cases, but on the other hand, it also shows that testing was still too unspecifically targeted [44], [45]. In April 2020, the German Federal Ministry of Health (BMG) published a first strategy paper that included information on PCR testing, the definition of contact persons and a guideline for the public health services (ÖGD) [46]. According to this, priority was given to testing symptomatic persons, their contacts, and every suspected case based on medical reasons. In addition, tested persons included hospital and nursing home staff caring for COVID-19 patients and particularly vulnerable groups in institutions such as hospitals, nursing homes, homes for the elderly, and institutions for people with disabilities [44]. The testing strategy was regularly updated during the course of the pandemic and adapted to the current situation [46]. To further clarify the issues of test eligibility, criteria and financing, the BMG published an “Ordinance on the Eligibility to Certain Tests” in June 2020 on the recommendation of the RKI [47]. However, a nation-wide regulation was not found, since eleven federal states had already developed their own concepts and a harmonization with the BMG regulation was not considered useful. It was argued that adaptation to local events/conditions must remain possible. Furthermore, it was maintained that the federal states could learn from each other. It remains dubious whether this federalism was helpful for infection surveillance [46].

In Bavaria, for example, all residents of the state, even those without symptoms, can be tested free of charge by licensed physicians since July 1, 2020. This offer has been criticized due to the consequently increased workload of the testing laboratories [48].

The national testing strategy was updated over the summer months, so that free tests were made available for returning travelers, and mandatory testing was imposed on people entering Germany from risk areas. With the end of the peak travel season, these regulations were lifted and the focus of testing shifted back to high-risk areas, such as hospitals or nursing homes [34].

In October 2020, the national testing strategy was expanded to include the use of antigen tests, which are primarily used in near-patient settings and are also approved as a self-test for the general population [49].

## Contact Tracing

Contact tracing, i.e., the determination of contacts, is distinguished from contact tracking, and does not include tracking the movement in real time or at all. The aim of contact tracing measures is to identify source cases and their contacts at an early stage, while respecting data protection, in order to break chains of infection and contain potential new infections [50]. Sufficient testing forms the basis for appropriate contact tracing. To prevent overburdening of healthcare systems in Germany, improve documentation of contact and risk encounters, and thus ensure better, individual contact tracing, there are several options for keeping a COVID-19 contact diary by using various smartphone apps [51], [52]. For example, in March 2021, the Luca app was released with the objective of providing fast and seamless contact tracing in cooperation with the GA via encrypted data transmission. Due to the ongoing COVID-19 pandemic and the dynamic developments of further surveillance tools, it is not possible to make a valid statement about the impact of these measures or present them exhaustively at this time [53].

Contacts are those persons who have been in contact with a confirmed SARS-CoV-2-infected person within the infectious time interval [54]. For symptomatically infected individuals, the infectious time interval extends from two days before the initial onset of symptoms to at least ten days after symptom onset. In case of asymptomatic infection and due to lack of information regarding the source and time of infection, the defined infectious time interval ranges from two days prior to testing to at least ten days after positive testing [50]. The recommendations for optimal behavior of contact persons differed depending on the classification into Category I (high risk of infection) or Category II (low risk of infection). In the case of an infection, information should be sent with the person’s name to the responsible GA, which will conduct a backward and forward investigation to identify sources of infection as well as potential routes of transmission. In the case of suspected contact with a SARS-Cov-2 variant of concern, it is not possible to shorten the quarantine period. For Category II contacts, the procedure is identical to Category I contacts in this regard [54].

In identifying contact persons, transmission routes, infections of multiple persons with the potential result of a large outbreak, significant potential exposure of at-risk groups or medical personnel, and suspected infection with a variant of concern of SARS-CoV-2 is prioritized. To immediately stop chains of infection in these cases, enhanced measures, contact identifications, and quarantine is immediately ordered [54].

## Cost-effective and facilitating measures through applications

In order to relieve the GAs and ensure infection surveillance, a number of technical innovations have been introduced. DEMIS, the *German Electronic Reporting and Information System for Infection Control*, was developed by the RKI in collaboration with other project partners and the support of the BMG, and is intended to ensure uniform, secure, rapid electronic reporting and information processing of positive SARS-CoV-2 pathogen detections nation-wide [41]. In this way, the previous manual workload of laboratories and GAs is to be reduced, so that infection protection measures can be initiated more quickly. In addition, SORMAS@DEMIS, the *Surveillance Outbreak Response Management and Analysis System*, was added to ease the burden on GAs during contact tracing [41] for faster identification of contact chains. 

The Corona Warning App (CWA) makes voluntary participation of the population in Germany in nation-wide surveillance possible for the first time [55], [56], [57]. Citizens have been able to download and use the app on their smartphones since June 16, 2020. The RKI, as the publisher of the app for the federal government, reports weekly data and information on the use or innovations of the CWA on its site. To date, 24.9 million downloads have been reported and the app has been made available in six different languages [58]. The security and legal underpinnings of the CWA have been examined from many angles [59], [60], [61], [62]. As of December, 90% of established labs are now connected to the app’s infrastructure. Those tested can view their results directly from the lab in the app via a QR code [52]. 

Based on theoretical modeling, Ferretti et al. [63] suggest possible inhibition of the COVID-19 pandemic if 80% of all smartphone users or 56% of the total population use the app. Urbaczewski and Lee [64] examined the effectiveness of CWAs in countries with mandatory app use and those with voluntary app use. They concluded that the CWA, despite voluntary use, can be effective in reducing the spread of COVID-19. However, with 24.9 million downloads, use in Germany remains below the effectiveness modeled at just 42% of all smartphone users and circa 30% of the total population [65]. Even though the numbers of downloads in Germany are still below the modeled effectiveness, this information alone by no means allows a conclusion about effectiveness. 

The CWA is a supplement to “manual” contact tracing by the GA, because it can also identify unconscious contacts that would otherwise not be detected. However, it in no way replaces contact tracing. Apps can document contacts as well as risk encounters and provide more accurate individual contact tracing. Capturing opaque spread of infection, such as on public transportation, as well as tracking it are difficult for public health departments [52]. 

The CWA in Germany was supplemented by the digital symptom diary *Climedo* [41]. Users of this app are asked daily about their health status when they are in quarantine, which would not be possible for the health authorities by telephone. 

## Emergency Room

Hospitals in Germany provide another source of surveillance data that can be used to draw conclusions about the course of the pandemic. In order to systematically record these data, multiple systems were developed and implemented in 2020. For example, the already existing system for nosocomial infection recording at the Charité Hospital in Berlin, in cooperation with the RKI, was supplemented in November 2020 by the module COSIK, *COVID-19-Surveillance in Hospitals*, as a pilot project [66]. COSIK collects data on the severity and progression of SARS-Cov-2 infections in outpatients and nosocomial hospitalized patients [66]. Another addition is the DIVI *Intensive Care Register* put forth by the RKI and *The German Interdisciplinary Association for Intensive Care and Emergency Medicine* (DIVI e.V.) [67]. This register is used to publish daily data on available intensive care beds and aims to identify the availability and bottlenecks of ventilation beds, to enable a regional and temporal comparison in real time. 

The variety of changes hospital surveillance has undergone during the COVID-19 pandemic is not described exhaustively in this article. Moreover, due to a cluster of articles in the literature search, the insights gathered from emergency room surveillance is presented here. Above all, as an enhancement of emergency room surveillance, the *SUMO* (*Surveillance Monitor*) program was developed by the RKI in close collaboration with the *ESEG* (*“Erkennung und Sicherung epidemischer Gefahrenlage*”) and published in April 2020 [68], [69]. The program collects data from emergency departments participating in the *Action Alliance for Information and Communication Technology in Intensive Care and Emergency Medicine* *(AKTIN)* registry. The collected data are accessible to the public, public health experts and decision makers and are made available for epidemiological research to identify and analyze clusters [70]. The numbers of admissions to German emergency departments, broken down by age group, three selected reasons for admission according to *CEDIS-PCL* (*Canadian Emergency Department Information System – Presenting Complaint List*) and the urgency assessment according to *ESI* (*Emergency Severity Index*) and *MTS* (*Manchester Triage System*) are published weekly in the situation report on the website of the RKI [68].

At the onset of the COVID-19 pandemic, a decrease in emergency department utilization of up to 40% compared to the mean in 2019 was observed [70]. This was followed by a steady, slow increase. In December 2020 and January 2021, a renewed decline was observed, but it was only 33% by comparison (RKI SitRep January 20, 2021 [68]). Two clusters were observed in the first weeks of the pandemic, but both were related to structural changes required by COVID-19 pandemic, such as the establishment of a COVID-19 outpatient clinic [70].

## Other aspects of the measures taken to date

The surveillance data collected are analyzed from various perspectives on a recurrent basis to assess pandemic activity. The uses of the data include estimates of severity, excess mortality, and socioeconomic implications.

The severity assessment of the novel virus SARS-CoV-2 had to be done in a very short time in 2020 to be ready as a basis for the measures to be taken. The WHO has developed criteria for the severity assessment of epidemics and pandemics caused by respiratory transmissible diseases. These criteria have been catalogued in the *Pandemic Influenza Severity Assessment Tool (PISA)* in collaboration with the RKI [71]. The Epidemic Potential, the Epidemiological Severity Profile and the Burden on the Health System are the indicators that form the basis for the criteria, which are examined usually using results from several parameters. The data collected in the ICOSARI Sentinel since 2015 is published weekly through the RKI’s influenza reporting, serving as a comparator for severity assessment. Five influenza waves have been recorded in the ICOSARI sentinel to date, forming a large data set as a baseline [72], [73]. Many modeling studies have been published [74], [75], [76], including by Sudharsanan et al., who described the case fatality rate (CFR) in nine countries in their July 2020 article and calculated a CFR of 9.3% in Italy, 7.4% in the Netherlands, and 0.7% in Germany [77]. This rate has not been confirmed over time, as 2% of all individuals who developed COVID-19 died in association with it in the first wave. Of the patients treated in hospitals, 22% died, compared to 6% of all patients in the ICOSARI sentinel [72], [75], [78]. Accordingly, a significantly higher mortality rate can be observed here. The RKI declares that in Germany, the same number of men and women fall ill with SARS-Cov-2, but men fall ill more severely twice as often and die more often [79]. This statement is not reflected in two age groups. In the whole of Germany, significantly more women between 35 and 59 years of age are tested positive than men in the same age group [80]. The discrepancy between the uniformity of the disease for women and men described by the RKI and the actual numbers in the middle age group must be investigated. Neither demographic data nor unemployment rates can explain this phenomenon. One hypothesis is that women in Germany are more willing than men to comply with the measures and take tests [81]. 

Surveillance data was used to identify early excess mortality associated with COVID-19. To do this, the Federal Statistical Office averaged the number of people who died in 2016–2019 and used that as the baseline mortality rate [82]. An advantage here is that the data can be presented in a very simple, transparent and low-threshold manner. A disadvantage is that the excess mortality from previous years is also included in the baseline mortality rate, it is no longer recognizable [82]. The data available to the Federal Statistical Office so far are raw data without plausibility and completeness checks. According to the analysis of these raw data, there was a significant increase in the number of deaths in Germany in the period from March 23 to May 3, 2020. The figures in the week from April 6 to 12 showed the highest number, with 15% more deaths compared with recent years. This was followed by an alignment with the average number of deaths, from 2016 to and including 2019, until August [82]. In August, probably due to a heat wave or/and scaled-back medical care during the first wave, there was an excess mortality of 7% [82], [83]. In the fall, excess mortality increased as infection rates increased from 5% in October, 12% in November to about 31% excess mortality in December [82], [84]. 

Research in recent decades in the field of socio-epidemiology has already identified strong correlations between risks of disease as well as death and the socioeconomic status of different population groups [85]. The lower the socioeconomic status of an individual is, the higher the risk of infection, also in terms of frequency and severity of the disease [85]. In Baden-Württemberg and Bavaria, states that have a very high overall socioeconomic status compared to the other states in Germany, significantly higher incidence figures were reported by mid-April 2020 than in socioeconomically weaker states [86]. This can be attributed, among other things, to the increased number of vacations in ski resorts (identified as outbreak sites at the beginning of 2020) which require certain financial resources, so that the incidence of infection at that time mostly affected socioeconomically more advantaged people [86]. Beginning in mid-April, however, the numbers flipped and regions with higher socioeconomic populations showed a decrease in incidence numbers, while an increase in COVID-19 cases was seen in low socioeconomic regions [86]. Data collected in the United Kingdom showed that populations from low socioeconomic environments had a 2.2-fold higher risk of testing positive for SARS-CoV-2 [85], [87]. Also, in the Williamson et al. study, these populations were disproportionately represented in intensive care units and had twice the risk of dying from COVID-19 compared to people from less deprived environments [85]. Other risk factors, such as heavy smoking, obesity, liver disease, and diabetes, also seem to favor severe disease progression of SARS-Cov-2 infection, which are common among socioeconomically less advantaged populations [85].

Based on the findings from the United Kingdom and the USA but also on the events and incidence figures from Germany, it is to be feared that socioeconomically weaker population groups have a higher risk of infection due to living and working conditions, and that the risk for a more severe course of COVID-19 disease is higher than in socioeconomically stronger population groups due to previous diseases and other behaviors [88], [89], [90]. In addition, the rapid spread of misinformation about COVID-19 through both social media and traditional media contributes to asymmetric information and promotes public uncertainty [91], [92].

## Limitations

Ad hoc literature reviews include limitations within a dynamic ongoing pandemic due to the sheer amount of research being done simultaneously. A scoping review like this offers a more open approach in contrast to a systematic literature review. The chosen timeframe entails a disregard for the changes that have taken place during the winter 2020–2021.

## Conclusion

In Germany, most changes to the existing infection surveillance measures were implemented after the first wave of the COVID-19 pandemic in the summer 2020. This should be critically questioned and scientifically investigated, as such a pandemic event was foreseeable and therefore ongoing considerations on pandemic preparedness should be taken into account. The reasons for the slow implementation needs to be investigated separately for each measure.

A clear change and innovation in the surveillance strategy is the broad involvement of the population, e.g., through the described apps and contact tracing. Despite all justified criticism, this also contains including the population in the testing strategy, for example with the help of “rapid tests”, which also requires comprehensive scientific monitoring. 

To draw conclusions about the impact different socioeconomic backgrounds have on COVID-19 cases in Germany, there may be a need to address data collection to better target and protect vulnerable segments of the population in the future. In addition, requirements that should also be addressed in preparation for future pandemics could include more rapid financial and staffing support for GAs to avoid excessive demand and associated inadequacies in infection surveillance.

## Notes

### Competing interests

The authors declare that they have no competing interests.

### Acknowledgements

We gratefully acknowledge Dr. Hani Kaba from our institute for his great support in searching for literature and Laura Koch and Lea Sommrey for critically proofreading and revising the manuscript.

### Authorship

Köster AM and Bludau A contributed equally as first authors.

Mardiko AA and Schaumann R contributed equally as last authors.

### Funding source

Funded by “NaFoUniMedCovid19“ (FKZ: 01KX2021) B-FAST

### Transparency declaration

Nothing to declare.

## Figures and Tables

**Table 1:**
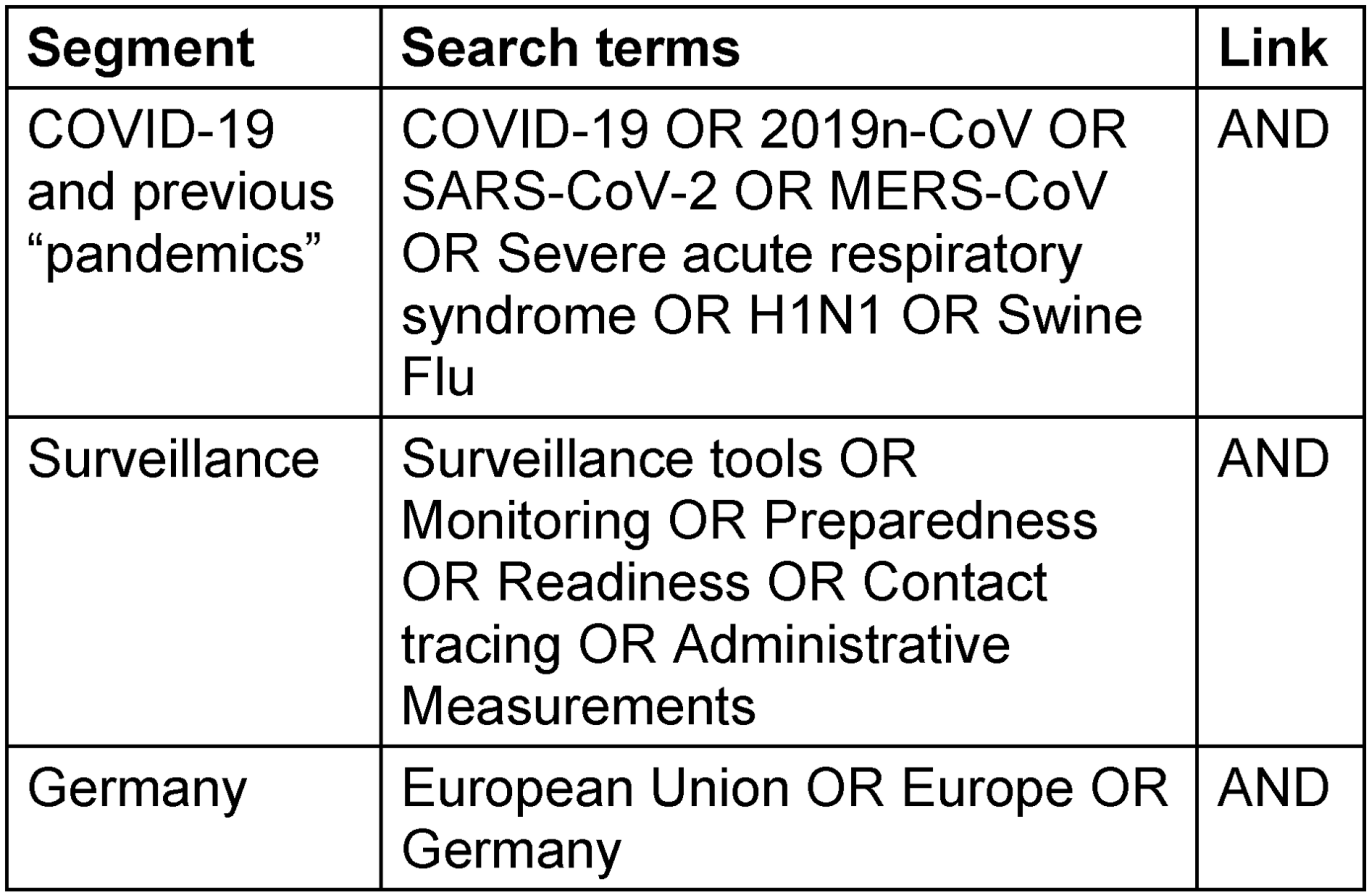
The search string used in the literature search
